# A novel method for quantitative analysis of subjective experience reports: application to psychedelic visual experiences

**DOI:** 10.3389/fpsyg.2024.1397064

**Published:** 2024-12-06

**Authors:** Sean Noah, Miranda Shen, Earth Erowid, Fire Erowid, Michael Silver

**Affiliations:** ^1^UC Berkeley Center for the Science of Psychedelics, University of California, Berkeley, Berkeley, CA, United States; ^2^Department of Neuroscience, University of California, Berkeley, Berkeley, CA, United States; ^3^Helen Wills Neuroscience Institute, University of California, Berkeley, Berkeley, CA, United States; ^4^Department of Psychology, University of California, Berkeley, Berkeley, CA, United States; ^5^Erowid Center, Grass Valley, CA, United States; ^6^Herbert Wertheim School of Optometry and Vision Science, University of California, Berkeley, Berkeley, CA, United States

**Keywords:** psychedelics, psychoactive substance, visual perception, visual effects, natural language processing, Erowid, large language model (LLM), subjective effects

## Abstract

**Introduction:**

Psychedelic compounds such as LSD, psilocybin, mescaline, and DMT can dramatically alter visual perception. However, the extent to which visual effects of psychedelics consistently vary for different substances is an open question. The visual effects of a given psychedelic compound can range widely both across and within individuals, so datasets with large numbers of participants and descriptions of qualitative effects are required to adequately address this question with the necessary sensitivity.

**Methods:**

Here we present an observational study with narrative self-report texts, leveraging the massive scale of the Erowid experience report dataset. We analyzed reports associated with 103 different psychoactive substances, with a median of 217 reports per substance. Thirty of these substances are standardly characterized as psychedelics, while 73 substances served as comparison substances. To quantitatively analyze these semantic data, we associated each sentence in the self-report dataset with a vector representation using an embedding model from OpenAI, and then we trained a classifier to identify which sentences described visual effects, based on the sentences’ embedding vectors.

**Results:**

We observed that the proportion of sentences describing visual effects varies significantly and consistently across substances, even within the group of psychedelics. We then analyzed the distributions of psychedelics’ visual effect sentences across different categories of effects (for example, movement, color, or pattern), again finding significant and consistent variation.

**Discussion:**

Overall, our findings indicate reliable variation across psychedelic substances’ propensities to affect vision and in their qualitative effects on visual perception.

## Introduction

Visual perceptual effects are hallmark features of psychedelic substances. LSD and psilocybin are two “classical psychedelics” that have been administered to human research volunteers (Carhart-Harris et al., 2016; Griffiths et al., 2006), and a great diversity of other psychedelic compounds serve as valuable research tools as well (Shulgin and Shulgin, 1997; Shulgin and Shulgin, 1991).

Based on their particular chemical structures, different psychedelic compounds have distinct effects in the brain. To the extent that different psychedelics also alter visual perception in characteristic ways, a comparison of physiological and qualitative effects across many psychedelics would be a powerful way to study the neural processes underlying conscious visual experience. However, how psychedelic visual effects vary for different substances is a largely open question. Anecdotally, visual effects often range widely across individuals even for a specific psychedelic compound, so a controlled experiment involving administration of psychedelics to human participants would likely not have the sensitivity to effectively answer this question. Here we address this question by conducting an observational study of narrative self-report texts, leveraging the massive scale of the Erowid dataset.

Erowid Center archives narrative self-reported texts of experiences with psychoactive substances, submitted by users and accessible to the public at its website.^1^ In our study, we analyzed experience reports associated with 103 different substances, with a median of 217 reports per substance. Thirty of the substances are generally recognized as psychedelics (some of which are also referred to as hallucinogens), while 73 served as comparison substances in our analyses and include sedatives, stimulants, herbs, and other drug classes. We designated substances in our study as psychedelic if they are characterized as such in the Erowid experience report dataset and if they either belong to tryptamine, phenethylamine, or lysergamide chemical classes or include compounds from these classes.

To quantitatively analyze these semantic data, we associated each sentence with a text embedding vector representation, mapping its semantic information to a mathematical form. The text-embedding-ada-002 model (OpenAI) (Greene et al., 2022) was used to generate vectors for each of the 2.2 million sentences in our text dataset. We then employed logistic regression to identify sentences describing visual subjective effects based on their vector representations. This analytical approach differs from previous text processing studies of the Erowid data set that relied primarily on word frequency-based or occurrence-based analysis methods (Mooseder et al., 2022; Nayak et al., 2021; Sanz et al., 2018; Zamberlan et al., 2018).

We observed that the proportion of sentences describing visual effects varies substantially and systematically across substances, even within the subset of psychedelic compounds. Next, we manually identified a group of categories of visual experiences by surveying the full set of visual effect sentences. For each substance, we calculated the proportion of visual effect sentences within each defined experience category, and we found that psychedelic compounds consistently differ in their profiles of visual effects.

Overall, our analyses demonstrate significant variation in psychedelic substances’ propensities to affect visual experience and other qualitative effects. Our findings also establish a new method for quantitative analysis and categorization of visual effects of psychoactive substances and other altered states of consciousness. The analysis method we describe here can be utilized in future studies to systematically characterize differences among psychedelic substances for various aspects of subjective experience. Our method also provides a foundation for future studies of psychoactive substances that relate physiological and biological measures to quantitative metrics of subjective experience, and our results indicate that the neurotransmitter receptor activity patterns that mediate psychedelic visual phenomena are multifactorial.

## Methods

### Erowid experience vault: subjective experience report dataset

Erowid Center is a nonprofit organization whose mission is to provide accurate and unbiased information about psychoactive substances freely to the public via its website, Erowid.org (Erowid, 1995–2023). Part of this mission involves informing website visitors about the acute effects of different psychoactive substances. The existing scientific literature on physiological and subjective effects is available on the Erowid website for a limited set of substances, but there are many more substances that people use recreationally, ceremonially, and medicinally whose effects have not been well characterized in experimentally controlled settings. Therefore, Erowid Center collects and maintains a publicly available archive of user-submitted text reports of subjective experiences with psychoactive substances.

Any Erowid website user may freely submit a text report describing their experiences with a psychoactive substance. Users may also submit reports of experiences that are related to psychoactive substances or other altered states of consciousness, such as dreams, meditation, drug testing, and law enforcement encounters. For our study, we excluded these reports from the analysis.

Erowid provides minimal guidelines and instructions for user-submitted reports, emphasizing well-written descriptive information about the user’s experiences, including mindset and setting, dosage and timing, physical and mental effects, preparation and intention, insights gained, and problems encountered. Erowid Center volunteers review submissions and screen out reports that are obviously fictional or exaggerated and/or do not provide useful information.

Researchers partnering with Erowid Center can access report text and metadata via an application programming interface (API). For our study, we used the Erowid API to download complete subjective report text for all substances associated with at least 100 distinct reports that were published between June 13, 1995 and May 22, 2023.

### Text preprocessing

We first preprocessed the text dataset in the Python programming language to prepare it for analysis. Initially, we employed the Beautiful Soup package (Richardson, 2007) to remove markup text that was used to format the reports for presentation on the Erowid website. Next, we separated each report into its constituent sentences by splitting the text at period characters. This procedure included exceptions for period characters between two numeral characters that we assumed denoted decimal points and for adjacent period characters that we assumed denoted ellipses. We associated each resulting sentence with a sentence identification number and a report identification number, along with a substance label. Reports that were associated with more than one substance in the Erowid metadata were excluded from the dataset.

The final dataset comprised reports for 103 substances (total of 39,586 reports; median of 217 reports per substance). The median number of sentences per report was 40, with a minimum of 1 and a maximum of 1,396. Table 1 summarizes the dataset at the level of individual substances. Substance names are presented in Table 1 and the following figures as they were entered in the Erowid experience report database verbatim. Supplementary Table 1 associates these Erowid database substance names with chemical names or other identifiers.

**Table 1:** Summary of Erowid experience report text dataset.

Substance	Number of reports	Mean sentences per report	Standard deviation	Minimum sentences per report	Median sentences per report	Maximum sentences per report
Cannabis	3,039	54.4	51.9	1.0	41.0	995.0
MDMA	2,507	56.7	58.6	1.0	42.0	979.0
LSD	2,488	80.0	82.8	1.0	59.0	1396.0
*Salvia divinorum*	2,450	54.4	44.0	1.0	44.0	498.0
Mushrooms	2,283	68.3	59.3	1.0	55.0	916.0
DMT	1,121	62.9	54.9	2.0	47.0	583.0
DXM	869	50.2	51.9	2.0	35.0	582.0
Mushrooms_P. cubensis	812	81.2	70.3	1.0	65.5	916.0
Cocaine	810	40.7	36.0	1.0	30.5	284.0
Ketamine	796	57.1	54.0	1.0	42.0	511.0
Morning Glory	647	59.4	50.7	2.0	47.0	520.0
Amphetamines	642	48.7	45.7	1.0	37.0	428.0
Kratom	594	39.6	44.4	1.0	30.0	428.0
2C-I	576	63.5	48.1	3.0	50.0	310.0
Methamphetamine	574	53.5	63.8	4.0	37.0	965.0
Syrian Rue	545	69.9	56.1	6.0	54.0	439.0
H.B. Woodrose	521	55.6	44.9	1.0	47.0	336.0
Nitrous Oxide	495	49.6	49.5	1.0	35.0	459.0
2C-B	469	61.1	47.2	4.0	50.0	389.0
2C-E	449	71.9	64.6	1.0	56.0	506.0
Heroin	430	50.1	43.8	2.0	39.0	399.0
Oxycodone	427	43.3	36.8	2.0	33.0	323.0
5-MeO-DMT	409	54.5	46.7	4.0	43.0	453.0
Alcohol	407	39.0	30.0	3.0	31.0	248.0
Pharms_Tramadol	403	31.3	32.3	2.0	22.0	388.0
Nutmeg	387	42.8	40.0	1.0	32.0	329.0
Pharms_Zolpidem	377	32.4	30.6	1.0	23.0	308.0
Diphenhydramine	375	48.6	37.5	1.0	39.0	228.0
Datura	353	53.6	42.9	1.0	40.0	303.0
Amanitas_A. muscaria	336	49.2	49.8	1.0	36.5	478.0
Hydrocodone	333	36.7	27.4	1.0	28.0	186.0
Pharms_Alprazolam	325	37.4	40.4	1.0	23.0	286.0
Caffeine	321	32.0	25.3	2.0	25.0	168.0
5-MeO-DiPT	320	49.8	38.2	3.0	37.0	249.0
4-AcO-DMT	316	76.0	53.7	7.0	62.0	361.0
AMT	313	59.0	50.1	4.0	44.0	410.0
Cacti_T. pachanoi	302	68.9	62.3	4.0	52.0	507.0
Alcohol_Beer_Wine	288	36.6	30.9	2.0	27.0	228.0
Alcohol_Hard	282	41.1	29.9	3.0	33.0	225.0
Pharms_Clonazepam	280	31.2	29.2	2.0	22.0	224.0
Kava	272	27.6	17.9	3.0	22.0	108.0
Pharms_Buprenorphine	256	33.0	33.7	1.0	24.0	277.0
Codeine	252	34.4	26.6	2.0	26.0	141.0
2C-T-7	246	54.2	42.3	1.0	43.0	329.0
25I-NBOMe	244	69.1	43.9	7.0	59.0	328.0
Dimenhydrinate	243	51.4	35.5	3.0	44.0	263.0
Methoxetamine	243	63.9	70.7	3.0	43.0	527.0
Pharms_Methylphenidate	240	47.0	57.0	2.0	31.0	428.0
Mimosa tenuiflora	236	81.6	65.7	7.0	63.5	583.0
DPT	235	62.8	44.6	2.0	50.0	224.0
Inhalants	220	36.6	60.3	3.0	27.0	853.0
GHB	217	40.4	39.8	5.0	28.0	325.0
Modafinil	210	34.2	32.4	3.0	25.5	266.0
Huasca Combo	203	76.7	52.6	9.0	61.0	338.0
Products_Spice-Like Smoking Blends	197	52.3	44.8	4.0	39.0	316.0
Methylone	194	58.4	49.2	3.0	46.5	305.0
Ayahuasca	193	103.5	86.4	7.0	81.0	568.0
Pharms_Bupropion	190	36.3	36.0	2.0	25.0	240.0
Pharms_Gabapentin	186	30.3	26.0	2.0	23.0	184.0
2C-T-2	177	54.2	47.2	6.0	39.0	280.0
Pharms_Venlafaxine	176	29.3	27.9	4.0	22.0	214.0
Methadone	164	35.2	37.0	4.0	23.0	259.0
Morphine	157	36.7	36.1	3.0	27.0	305.0
4-Methylmethcathinone	156	52.5	84.5	4.0	35.0	995.0
Tobacco	154	35.4	37.9	3.0	22.5	241.0
MDA	148	52.4	30.9	6.0	44.0	158.0
Calea zacatechichi	147	31.3	24.6	3.0	25.0	178.0
Poppies_Opium	146	41.7	37.2	7.0	32.0	299.0
*Banisteriopsis caapi*	145	82.1	63.6	4.0	65.0	443.0
Melatonin	143	22.7	19.0	1.0	16.0	134.0
Pharms_Paroxetine	142	31.1	27.1	2.0	22.0	195.0
Crack	140	37.4	33.0	1.0	27.0	238.0
Pharms_Quetiapine	137	28.3	27.0	3.0	19.0	148.0
Cannabis_Hash	137	50.3	43.6	7.0	37.0	248.0
Lotus_Lily_Nymphaea nouchali var. caerulea	137	33.8	24.7	2.0	26.0	133.0
5-MeO-AMT	137	52.2	40.2	2.0	43.0	269.0
Pharms_Pregabalin	136	28.2	21.3	3.0	21.5	107.0
Absinthe	133	30.0	21.0	3.0	25.0	119.0
25C-NBOMe	128	66.8	50.1	7.0	52.0	281.0
Smarts_Phenibut	125	43.1	38.1	4.0	34.0	263.0
Damiana	124	23.1	20.2	4.0	18.0	119.0
Pharms_Diazepam	124	41.7	38.7	3.0	30.5	224.0
1P-LSD	122	74.7	58.2	2.0	62.5	348.0
PCP	122	44.8	30.7	6.0	36.0	157.0
Catnip	121	22.2	17.1	3.0	17.0	133.0
Valerian	118	25.8	25.6	2.0	19.0	177.0
4-HO-MET	116	73.1	48.1	1.0	61.0	275.0
Cacti_T. peruvianus	115	87.5	114.2	4.0	63.0	977.0
Pharms_Fentanyl	114	33.4	26.3	2.0	26.0	141.0
2C-C	114	59.1	51.7	6.0	39.5	294.0
5-MeO-MIPT	114	61.8	45.2	8.0	47.0	225.0
MDPV	113	39.8	36.6	5.0	26.0	185.0
Etizolam	113	41.7	48.0	3.0	29.0	307.0
Pharms_Lorazepam	109	38.1	36.0	2.0	26.0	184.0
JWH-018	106	50.4	41.6	5.0	40.0	246.0
Sceletium tortuosum	104	30.5	30.0	6.0	23.0	201.0
Wormwood	104	27.7	19.4	3.0	25.5	119.0
DOC	104	87.5	64.8	7.0	69.0	373.0
Pharms_Sertraline	104	32.6	27.5	5.0	24.0	156.0
Mescaline	104	86.8	92.7	8.0	65.0	654.0
Brugmansia	103	58.1	40.7	2.0	44.0	162.0
Piracetam	103	37.6	41.9	4.0	26.0	369.0
Huasca Brew	102	85.3	64.6	13.0	66.0	362.0

Substances are listed in descending order of the number of reports for each substance.

### Text embedding vectors

To quantitatively analyze the semantic content of the experience report texts, we associated each sentence in the dataset with a text embedding vector. A text embedding is a mapping from character strings to vectors such that the semantic similarity of two strings is related to the mathematical similarity of the associated vectors. For sentence embedding, two sentences with similar meanings (e.g., “I sauteed the tofu” and “I braised the bean curd”) would be separated by a smaller Euclidian distance in vector space than two sentences with more different meanings (“I sauteed the tofu” and “The president enjoyed my cooking”).

For each sentence in the text dataset, we generated a corresponding embedding vector using the text-embedding-ada-002 model (OpenAI) (Greene et al., 2022). Text embeddings were computed with the OpenAI API over a period from July 10, 2023, to July 23, 2023. The text-embedding-ada-002 model associates any input text string with a 1,536-dimensional vector, and at the time of our analysis, it was the highest performing text embedding model available from OpenAI (Greene et al., 2022).

### Visual effect sentence classifier

Erowid experience reports are unconstrained narrative reports that often contain information about a psychoactive substance user’s mindset and setting, context and motivation, methods of preparation and administration, etc., along with descriptions of various subjective effects. In order to systematically compare visual subjective effects across substances, we needed to identify descriptions of visual subjective effects in narrative text, but our dataset was too large to allow manual evaluation of every sentence. We therefore developed a logistic regression classifier model to detect sentences that describe visual effects, based on their vector embeddings.

First, we created a set of labeled sentences to train the logistic regression model. We randomly sampled 10,000 sentences, without replacement, from the report dataset. For each sentence, we performed an OpenAI GPT-4 language model API call to label visual effect sentences. This GPT-4 API call used the following system prompt: “You are a model that identifies effects of psychoactive substances on visual experience (1) or not (0). Classify the following sentence. Respond only with 1 or 0. A 1 means that the sentence explicitly describes a visual effect.”

The user prompt for each API call was one of the 10,000 sampled sentences, generating a 1 or a 0 response for each of the sampled sentences. The API call temperature was set to 0.0 to minimize the possibility of unexpected (“creative”) responses to the prompt. Of the 10,000 sampled sentences submitted to GPT-4 for labeling, 1,231 (12.31%) were labeled as visual effect sentences, and 8,769 (87.69%) were labeled as not visual effect sentences.

To check the quality of the automated labeling procedure, we performed a manual review on a random sample of 100 of the sentences labeled by GPT-4 as 0 and 100 of the sentences labeled as 1. One of the investigators (S.N.) made a subjective judgment about whether each of these sentences were visual effect sentences or not, without knowledge of the labels assigned by GPT-4. All 100 of the sentences that were labeled as “not visual effect” by GPT-4 were classified the same way by the manual review. However, 18 of the sentences that were labeled as “visual effect” by GPT-4 were classified as “not visual effect” by the manual review. We therefore manually reviewed all 1,231 sentences labeled as “visual effect” by GPT-4, correcting the labels where necessary to reduce the false positive rate of our subsequent classifier training on the full data set. This resulted in a change of labels of 244 “visual effect” sentences to “not visual effect,” with a final count of 987 “visual effect” sentences and 9,013 “not visual effect” sentences.

We then used the Scikit-learn (Pedregosa et al., 2011) Python package to build a logistic regression classifier for our labeled training data. First, we added the corresponding embedding vectors to the labeled sentence dataset. We then trained the logistic regression on the embedding vectors, with each of the 1,536 dimensions as an input variable, and the corresponding sentence label (“not visual effect” or “visual effect”) as the outcome variable.

We used an 80–20% train-test split procedure and undersampled the training set such that the number of “not visual effect” sentences matched the number of “visual effect” sentences (987 sentences). The prediction accuracy of this training procedure was 89.3%. Prediction accuracy is defined as the number of sentences for which the labels produced by the logistic regression classifier matched the labels in the training set, divided by the number of sentences in the test set.

We then used the trained classifier to predict the probability that each sentence in our full Erowid sentence dataset was a visual effect sentence. We manually reviewed a sample of classified sentences to assess whether the predicted probabilities generated by the classifier corresponded to our own judgments of “visual effect” versus “not visual effect” sentences.

Example sentences and their associated classifier probabilities are displayed in Table 2. We observed that the prediction probability was generally well correlated with our own certainty about whether a sentence described a visual effect. Based on our review, we chose a prediction probability threshold of 0.75 to classify a sentence as a visual effect sentence, aiming to limit false positives. The total number of visual effect sentences above this prediction probability threshold was 143,520, corresponding to 6.52% of the total number of sentences in the full data set.

**Table 2:** Examples of Erowid subjective report sentences and visual effect logistic regression prediction probabilities.

Sentence	Probability
Would I repeat the experience?	0.12
It’s felt like ten seconds and it’s felt like two years.	0.24
But the chill of winter is still in the air- old father death breathing rebirth into the spring breeze.	0.33
There was an incredible sense of loneliness and intense pain there.	0.44
I feel like I am walking crazily, I have to concentrate really hard to feel like I am walking normally.	0.51
The metal poison feeling was gone, but I was think, heavy and separated from my body, like watching myself move but unable to control or know where I would go.	0.57
He started to bark and as he did his voice would start to echo and as my sense took in the noise I began to dissociate.	0.61
I felt an extreme apartness from everyhting around me except the natural world.	0.62
I looked to my left where they were banging but I could not see them at all.	0.68
But the rest was an infinity of thick white feathers, and I had a strong connection to each of them.	0.69
I would look at a painting I normally woulnd’t look twice at (in fact one I looked at I had never looked at for the previous 20 years we had it) and was mystified.	0.71
As this started to happen I lost myself in almost a separate reality.	0.72
Then, some very weak hallucinations - fond memories of an unknown place that really did not exist - came up in my head and I layed there, remembering them for what felt like the entire night.	0.74
again if I look to the side when I’m moving forward I get extremely disoriented.	0.77
Because the bizarre visual features seemed to make it worse, I turned off the light and got into bed.	0.82
I also think now that many of my visual problems stemmed from an effect of the drug that made me unable to see pictures or visual input as a ‘whole’ picture (as we usually perceive what we are looking at) instead of a collection of many millions of small details.	0.82
The rest of the day consisted of seeing the sky change colors, water boiling and burning and sometimes seeing a man with a knife yelling at me.	0.84
From there my eyes really changed.	0.85
Everything in my vision became a circular blur, a tunnel, around everything but a tiny hole where my eyes were focused.	0.97

Sentences are listed in ascending order of prediction probability. A probability of 0 indicates that the model is certain that the input sentence does not describe a visual effect, and a probability of 1 indicates the opposite. We designated a probability of 0.75 as the threshold for classifying whether a sentence is a visual effect sentence or not.

**Figure 1:**
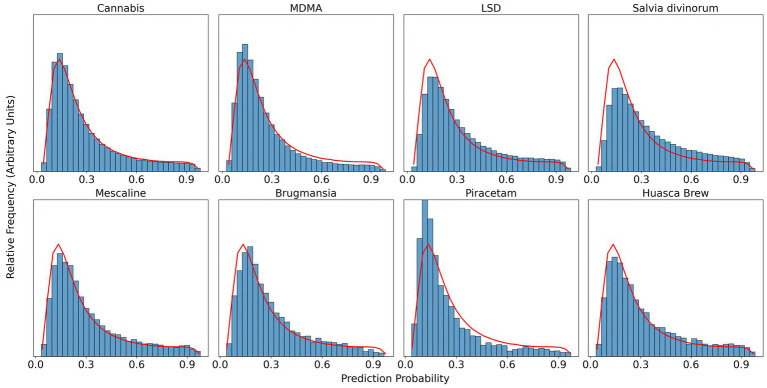
Histograms of the relative frequency distribution of prediction probability values for selected substances. For each subplot, prediction probability values are represented along the horizontal axis, and relative frequency is represented along the vertical axis. Because the different substances have different numbers of sentences in their total experience report data, the vertical axis scales are normalized so that the maximum frequency value is plotted at the top of each subplot. The red line in each subplot depicts the overall distribution of prediction probabilities for the entire dataset. Prediction probability values were calculated by the trained logistic regression classifier model for each sentence in the dataset.

In Figure 1, we display the prediction probability distribution for all sentences in the dataset and for a selection of individual substances. In general, most of the sentences have a prediction probability below 0.5. This was expected, given that Erowid reports often describe many more aspects of a psychoactive substance experience than just visual subjective effects. We display the prediction probability distribution for the top four and bottom four number of reports (substances needed to have at least 100 reports in the data set to be included in our analysis) (Figure 1). We observed that the general shape of the probability distributions is similar across substances, with most variation across substances occurring in the upper end of the distribution. This variation is further explored below.

### Categorizing visual effects

In this study, we assessed whether experience reports from users of psychedelic and other psychoactive substances consistently vary in their descriptions of visual effects. To conduct this analysis, we first extracted those sentences describing visual effects from the original subjective reports using the trained logistic regression classifier. We created a new visual effect sentences dataset consisting of all sentences whose classifier prediction probability was at least 0.75, along with their substance labels and their embedding vectors. We then used the embedding vectors to quantitatively analyze the distributions of types of visual effects across substances.

Embedding vectors translate semantic similarity of sentences into mathematical distance in vector space. We therefore used the embedding vectors to quantify, for each substance, the proportion of sentences that describe particular categories of visual effects. To determine the categories of visual effects to be analyzed, we surveyed the full visual effect semantic space by projecting the embedding vector dataset into two dimensions using uniform manifold approximation and projection (UMAP), a dimensionality reduction method optimized to maintain the global and local structure of high-dimensional data that have nonlinear variation (McInnes et al., 2018).

**Figure 2:**
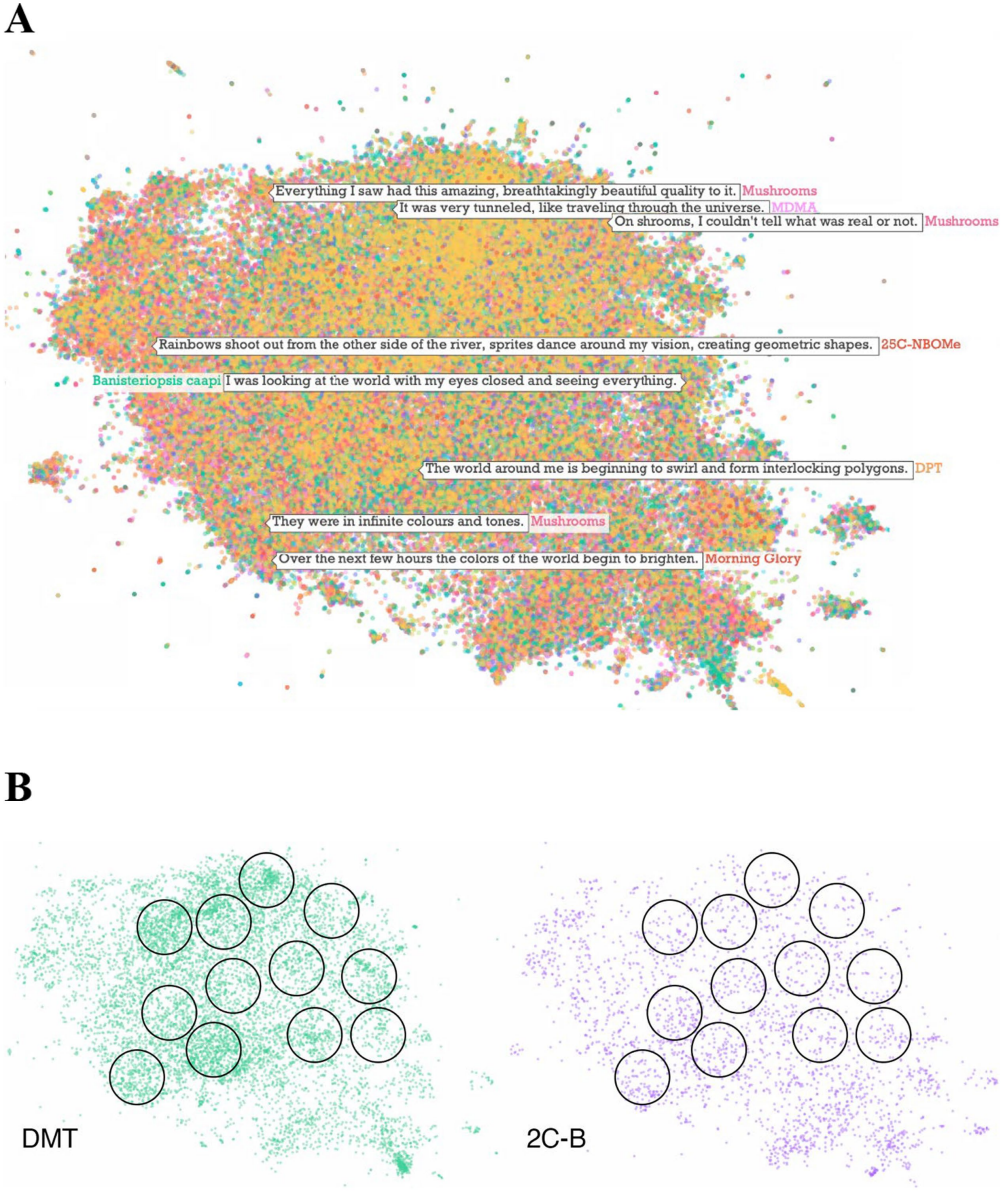
**(A)** Two-dimensional UMAP projection of all visual effect sentence embedding vectors. Each point corresponds to a single visual effect sentence, and colors denote different substances. The locations of some example sentences are indicated with arrows. **(B)** Illustration of visual effect categorization and calculation method. Seed sentences (listed in Table 3) were created to represent different regions of the visual effect vector space. Each visual effect was defined as the area within a threshold distance of a seed sentence vector. The proportions of visual effect sentences that fell within each category were then compared across substances. The black circles overlaid on the 2-dimensional point clouds demonstrate our procedure for categorizing visual effect sentences by calculating distances from seed sentence vectors (the center of each circle) and setting a distance threshold. Note that the analysis was conducted in the original 1,536-dimensional vector space, and the two-dimensional projection with overlaid circles/visual effect categories shown here is for illustration purposes.

The UMAP 2-D projection of the embedding vectors facilitates assessment of how different visual effect sentences are semantically related to one another and identification of prototypical visual effect sentences representing distinct regions of the visual effect sentence space. Specifically, one of the authors (SN) surveyed the 2-D UMAP of visual effect sentences by densely sampling and reading sentences across the projection to explore the semantic space. Based on this survey, the same author (SN) then manually generated prototypical sentences to reflect common types of visual effect descriptions encountered. This process is illustrated in Figure 2, and the prototypical sentences we generated as seed sentences for further analysis are listed in Table 3.

**Table 3:** Seed sentences for visual effect categorization analysis.

Visual effect category	Seed sentences used for analysis
Pattern	I saw geometric patterns.
	I saw kaleidoscopic patterns.
	I saw fractals.
	I saw patterns.
	I saw repetitions.
Movement	I saw things breathing.
	I saw things morphing.
	I saw things drifting.
	I saw things waving.
	I saw things melting.
	I saw things flowing.
	I saw tracers and trails.
Color	I saw colors.
	I saw colors changing.
	Colors were beautiful.
	I saw rainbows.
	I saw colors I had never seen before.
Entities	I saw entities.
	I saw aliens.
	I saw angels.
	I saw demons.
	I saw elves.
	I saw God.
Global visual alterations	My vision was blurry.
	My vision was sharpened.
	Everything looked dark.
	Everything looked bright.
	I saw a bright light.
Distortion	Everything was distorted.
	I saw things in my peripheral vision.
	I saw a distorted face.
	I saw cartoons.
	I saw things get bigger.
	I saw things get smaller.
	I saw things change in size.
Affect	I saw beauty.
	What I saw scared me.
Other	I experienced synesthesia.
	I saw a tunnel.
	I saw things when I closed my eyes.
	I could not tell what was real.

We generated these sentences as being prototypical for the different categories of visual effects that we observed in the full set of visual effect sentences, and we then used them as seed sentences for analysis of visual effect categories.

**Figure 3:**
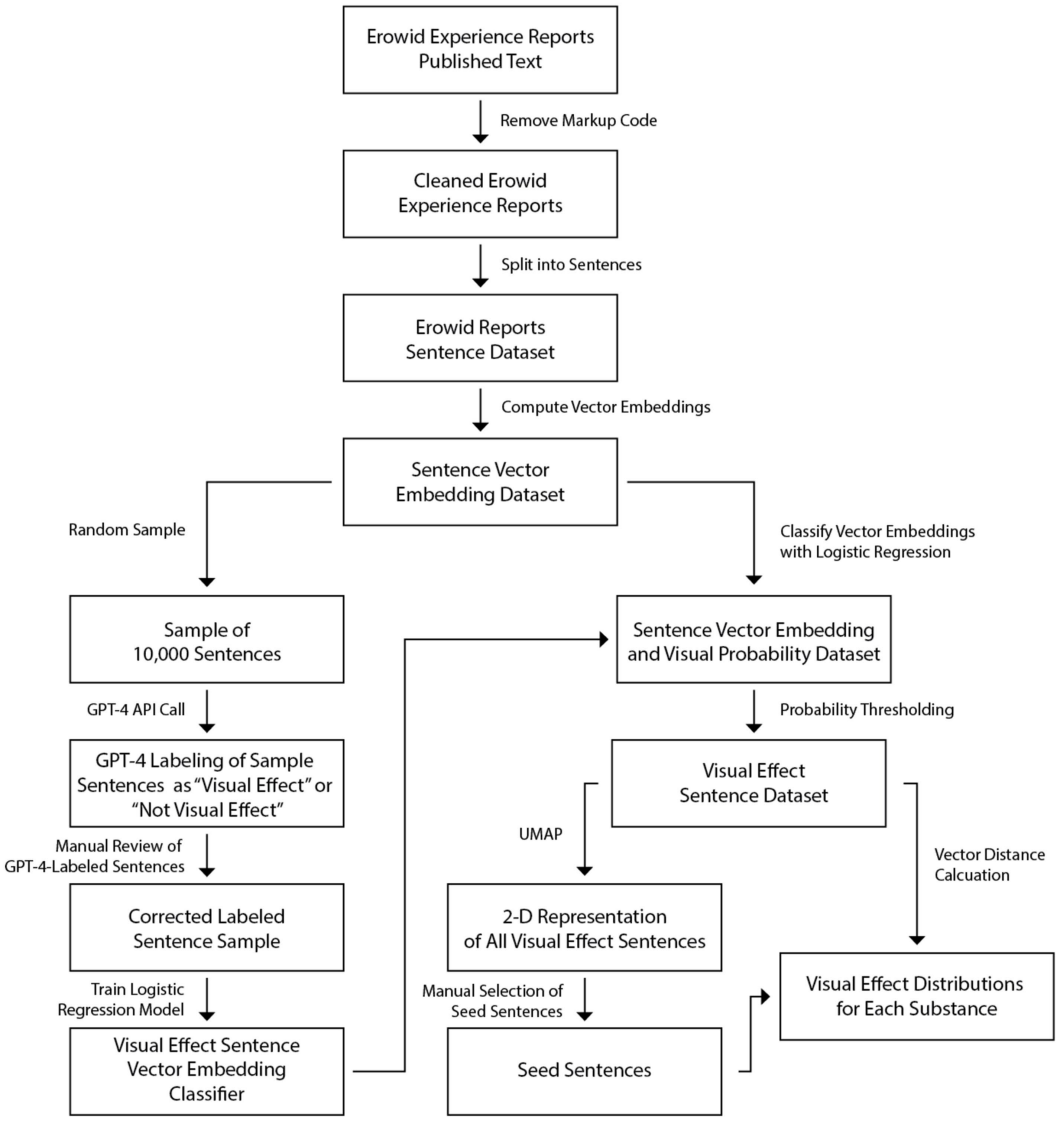
Flowchart of method for calculating visual effect distributions across substances from the Erowid experience report dataset.

To quantify any given substance’s likelihood of causing different visual effects, we calculated the proportion of that substance’s visual effect sentences that contained embedding vectors within a distance threshold of each seed sentence. We defined a distance threshold of 0.55, based on a survey of subjective report sentences and their vector distances from the seed sentences. We determined that this threshold value is the approximate distance above which sentences are no longer semantically similar enough to the seed sentence to justify being labeled as an instance of that category of visual effect. Figure 2 illustrates this distance calculation method for a two-dimensional UMAP projection (although the quantitative analysis involves computing distance over all 1,536 dimensions). Figure 3 illustrates the full analysis method with a flowchart.

To test for statistical significance of differences among psychedelic substances in their profiles of visual effect sentence categories, we performed a permutation test using a statistic that measures the differences across substances in their respective associations with seed sentences. This test statistic *T* quantifies the average inter-substance variability in relation to each seed sentence. Specifically, for each seed sentence, the mean absolute difference between the sentence proportion values was computed for all pairwise comparisons of substances, thereby quantifying the dispersion of each substance’s association with that seed sentence. This process was repeated for each seed sentence, and the value of the test statistic is the overall mean of these differences. This measure summarizes the variation among substances in their associations with the spectrum of visual effect categories.

Formally, *T* is defined as:


$$T=\frac{1}{\left|S\right|}\underset{s\in S}{\sum }\left(\frac{2}{|D|\left(|D|-1\right)}\underset{d1,d2\in D;d1\neq d2}{\sum }|V\left(s,d1\right)-V\left(s,d2\right)|\right)$$

Where:

- *S* is the set of all seed sentences,
- *D* is the set of all substances,
- *V*(*s*,*d*) represents the value (the proportion of subjective report sentences below the distance threshold) for seed sentence *s* and substance *d*,
- ∣*S*∣ and ∣*D*∣ represent the number of seed sentences and substances, respectively.

In this equation, the inner sum represents the mean absolute difference between all pairwise comparisons of substances for seed sentence *s*. The outer sum is the average of these mean differences across all seed sentences.

We created seed sentences that reflected categories of visual effect sentences that we frequently encountered while manually reviewing the list of visual effect sentences, arranged by their semantic similarity in the 2-D UMAP projection. Therefore, the generation of seed sentences was a top-down, rather than data-driven, process. In future work, a data-driven method for identifying visual effect categories without manual classification would allow for a more objective analysis of systematic differences among psychedelic substances’ visual effects. However, for the present study, we reasoned that manually identified visual effect categories that exhibited statistically significant differences would allow testing for differences in the visual subjective effects among psychedelic substances.

## Results

**Figure 4:**
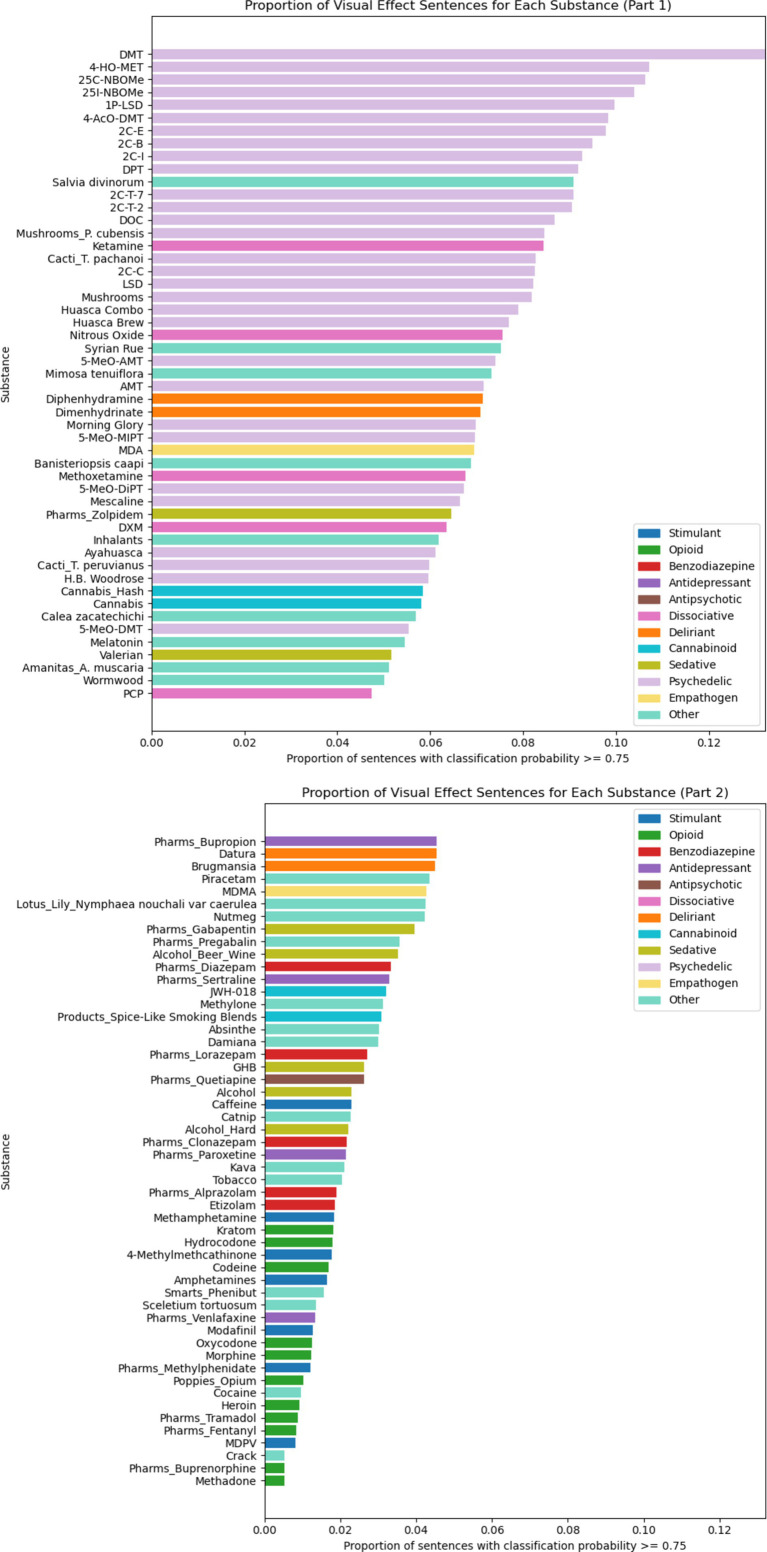
Proportion of visual effect sentences for each psychoactive substance. Substances are color coded by drug class.

We observed that the proportion of visual effect sentences varies significantly by substance (Figure 4). Using a chi-square procedure, we rejected the null hypothesis that across all sentences in the report dataset, substance and prediction probability vary independently, X^2^ (114, *N* = 2,246,254) = 72,912, *p* < 0.001. The strength of association between substance and prediction probability, measured by Cramér’s *V*, was 0.180, indicating a small-to-moderate association. Psychedelic compounds tend to have a much higher proportion of visual effect sentences than any other drug category. We performed a two-sample t-test comparing the proportion of visual effect sentences for psychedelic versus non-psychedelic substances and found that psychedelic compounds have a significantly higher proportion of visual effect sentences (*p* < 0.001).

**Figure 5:**
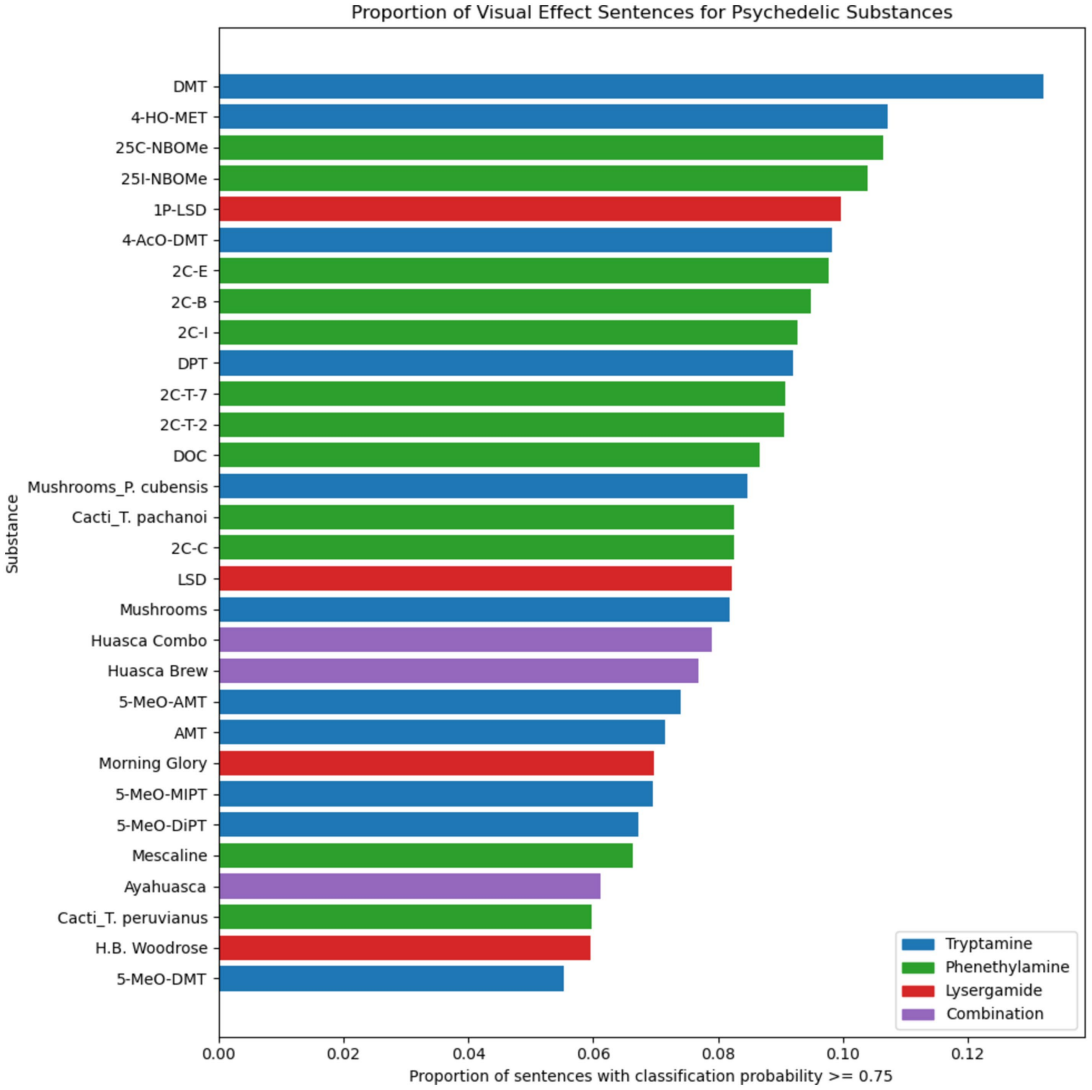
Proportion of visual effect sentences for each psychedelic substance. Psychedelic substances are color coded by drug class.

The proportion of visual effect sentences also varied significantly by substance when analyzing only psychedelic substances (Figure 5), X^2^ (29, *N* = 931,858) = 5,771, *p* < 0.001. The strength of the association between psychedelic and prediction, measured by Cramér’s *V*, was 0.078, indicating a small association.

**Figure 6:**
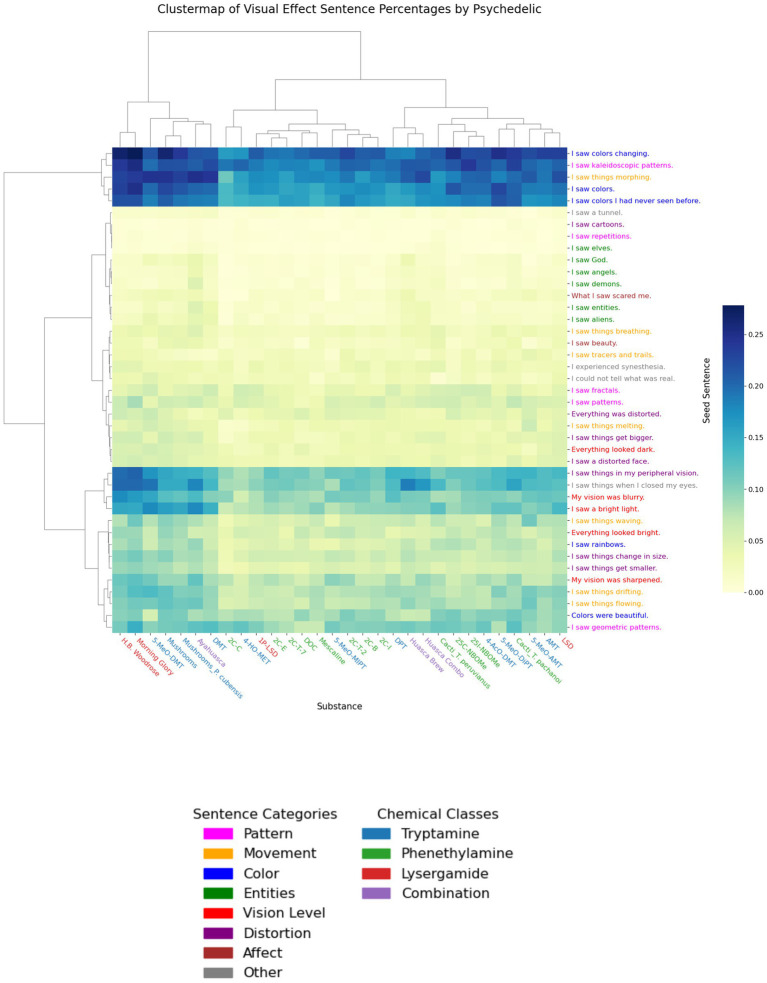
Clustermap of visual effect sentence proportions for each psychedelic substance. For each seed sentence, we calculated the proportion of visual effect sentences for each psychedelic substance that fell within a distance threshold in embedding vector space. This proportion quantifies the strength of association between report sentences and a given seed sentence and is visualized using the color map on the right of the figure. This map combines heatmap and dendrogram methods, displaying the hierarchical clusters among substances and visual effect sentences. In the dendrograms, the total branch distance between two leaves (seed sentences or substances) indicates dissimilarity between those two leaves. Substance labels are color coded by psychedelic chemical class, and seed sentence labels are color coded by sentence category.

The analyses displayed in Figures 4, 5 demonstrate reliable variation of the proportion of visual effects sentences across all substances and across psychedelic substances. In addition, we conducted a categorization analysis (see Methods) that showed that the proportions of different categories of visual effects vary across psychedelic substances. These results are displayed as a clustermap of proportions of categories of visual effect sentences, with seed sentences and substances arranged according to their dendrogram distances (Figure 6). Each leaf of the dendrogram represents one observation (a row or a column of the heatmap). Branches connect the leaves, and the number of branch crossings that must be traveled between two leaves represents the dissimilarity between those two leaves within the category (sentences or psychedelic substances) (Figure 6).

We defined dissimilarity between two substances or two seed sentences as the Euclidean distance between their proportion vectors. The resulting clustermap reveals three distinct groups of seed sentences over all substances: relatively high, moderate, and low proportion values (Figure 6). These three clustermap groups do not map directly onto the seed sentences categories that we manually determined. For example, the high proportion clustermap group contains Color, Movement, and Pattern seed sentences, but these sentence categories are also found in the other two groups (Figure 6).

By visual inspection, the longest branches in the dendrogram for the seed sentences can be used to distinguish three groups. The clustering of seed sentences into these three groups suggests that variation in the overall proportion values across substances is the most important factor for clustering the seed sentences. In contrast, the dendrogram for the psychedelics does not reveal clearly distinct substance groups to the same extent as the dendrogram for the seed sentences (Figure 6). The substance dendrogram suggests that there are three major clusters of psychedelic substances, but the lengths of the branches that separate the three substance clusters are shorter than the corresponding branches for the three seed sentence clusters, suggesting that the dissimilarity of proportion values in the three clusters is less for the substances than it is for the seed sentences.

We defined a test statistic *T* that quantifies the extent to which psychedelic substances differ from one another in the strength of their associations with seed sentences (see Methods). For the dataset of visual effect sentences, *T* = 0.0165. We then created a null distribution by shuffling the substance labels in this dataset and recalculated *T* for the proportion values derived from the shuffled-label dataset.

We performed this shuffling and *T* calculation procedure 10,000 times to generate a distribution of *T* values that could be expected under the null distribution that there is no association between substance and visual effect profile. We then calculated the *p*-value as the proportion of permuted *T* values that were greater than or equal to the observed *T* value. This *p*-value was less than 0.001, indicating significant consistent variation across psychedelic substances in their relationships with seed sentences.

## Discussion

We found that experience reports for different psychedelic substances vary in their proportions of different categories of visual effect sentences (Figure 6), suggesting that the profiles of visual effects for a given psychedelic are multifactorial. This would provide support for the patterns of receptor activation underlying the perceptual effects of psychedelics also being multifactorial, rather than being reducible to action at a single type or subtype of neurotransmitter receptor.

The 2A subtype of serotonin (5-hydroxytryptamine, or 5-HT) receptors (5-HT_2A_ receptors) has been proposed to mediate the subjective effects of psychedelics. Classical psychedelics like DMT, LSD, psilocybin, and mescaline are sometimes termed serotonergic psychedelics because of their affinity for 5-HT receptors, particularly the 2A subtype (Nichols, 2016). The 5-HT_2A_ receptor subtype has been described as necessary for some effects of classical psychedelics, including subjective ratings of complex visual imagery and increased visual cortical excitability (Kometer et al., 2013; Preller et al., 2018; Quednow et al., 2012). In addition, 5-HT_2A_ receptors are densely expressed in visual cortex (Beliveau et al., 2017).

Our results are consistent with the notion that activation of the 5-HT_2A_ receptor does not solely dictate psychedelic visual phenomenology. If psychedelics varied only in their binding affinities or activation levels of 5-HT_2A_ receptors, the intensities of their effects could be expected to vary across psychedelic substances as a function of dose. However, in this case, the profiles of subjective effects would be more consistent across different psychedelic substances, especially for two substances with similar binding affinities and functional activations of the 5-HT_2A_ receptor. In contrast, our results suggest that different psychedelics have characteristically different distributions of visual subjective effects and that there is a statistically significant association between these profiles of effects and the corresponding substances.

Results from studies employing the 5-HT_2A_ receptor antagonist ketanserin have been interpreted to mean that activation of this receptor subtype may be necessary for the characteristic psychoactive effects of psychedelics (Kometer et al., 2013; Preller et al., 2018; Quednow et al., 2012). However, several non-psychedelic 5-HT_2A_ receptor agonists have been identified. For example, lisuride and ergotamine are analogs of LSD that have been previously described as non-psychedelic because their subjective effects do not resemble those typically associated with psychedelics (González-Maeso et al., 2007; Pieri et al., 1978). However, these drugs are 5-HT_2A_ receptor agonists at levels comparable to those of their psychedelic congeners, as assessed by functional measures of receptor activation (Bonhaus et al., 1997; Egan et al., 1998). The existence of non-psychedelic 5-HT_2A_ receptor agonists indicates either that 5-HT_2A_ receptor activation alone is insufficient to cause visual effects, or that there is a threshold of receptor activation that must be reached to produce psychedelic effects in a particular signaling pathway or population of neurons (Wallach et al., 2023).

Furthermore, recent evidence indicates that ketanserin, in addition to being an antagonist for the 5-HT_2A_ receptor subtype, also has moderate affinity for the 5-HT_2C_ receptor subtype, as well as moderate to high affinity for several adrenergic and histamine receptors (Casey et al., 2022). Therefore, the profile of receptor activation across multiple serotonin receptor subtypes and other types of neurotransmitter receptors, rather than just the level of activation of 5-HT_2A_ receptors, is likely to determine the full set of subjective effects of any given psychedelic compound.

We observed that the proportion of visual effect sentences in the full dataset of experience reports varies significantly across all substances (Figure 4) and for the category of psychedelic substances (Figure 5). This variation represents differences across substances in their propensity to cause visual effects relative to other types of effects. Notably, psychedelics had significantly greater proportions of visual effect sentences than non-psychedelic substances, even though they also generally tended to have greater number of sentences per experience report in general (Table 1). Erowid experience reports are unconstrained and unprompted, and it is therefore likely that description of an effect in a given report reflects how salient or memorable that effect was for the report’s author.

Our findings of multifactorial visual effect profiles and varying propensities to cause visual effects across psychedelics, together with pharmacological evidence of different psychedelic compounds’ varying binding affinities across neurotransmitter receptor types (Jensen and Roth, 2008), suggest the importance of activation of multiple types of neurotransmitter receptors to account for the phenomenology of any given psychedelic compound. This possibility has been previously described in the literature. For example, it has been hypothesized that activation of 5-HT_1A_ receptors may have larger effects on central visual processes than 5-HT_2A_/_2C_ receptor activation (Nichols, 2000). However, there is currently no clearly established link between receptor types/subtypes and propensities for visual psychedelic effects or types of visual effects. Other possible mechanisms that could lead 5-HT_2A_ agonists to have differing visual effects include agonist-directed trafficking (Berg et al., 1998), various forms of biased agonism, and activity of metabolites of ingested psychedelic substances.

Based on Cramér’s *V* values, there was a small-to-moderate effect size for the variation of proportion of visual effect sentences across all substances, and a small effect size when the analysis was limited to psychedelic substances. The larger effect size in the all-substance analysis is driven by the varying propensities of different drug classes to cause visual effects, with psychedelics being the most likely, and opioids being the least likely, to cause visual effects (Figure 4).

Our study demonstrates the utility of analysis of large-scale narrative self-report data in the study of the phenomenology of psychoactive substances. Individual substances can cause wide ranging and highly variable visual effects both within and across individuals, so large and high-quality data sets (such as Erowid’s Experience Vaults) are needed to accurately derive the visual effect probability distributions for different substances and to have the statistical power to meaningfully compare them to one another. To our knowledge, our study is the first to quantitatively demonstrate that different psychedelic substances result in different types of visual experiences.

The approach of quantitatively studying semantic text data that we describe here represents a methodological advance, combining the strengths of natural language processing and qualitative analysis of text. We used a text embedding model (text-embedding-ada-002; OpenAI) (Greene et al., 2022) to map sentences to vectors in a way that translates semantic similarity among sentences to mathematical similarity among vectors, and we used the GPT-4 large language model (OpenAI) to automate the creation of a labeled training set for our sentence embedding vector classifier.

By combining text embedding and large language models, we identified visual effect sentences in the Erowid dataset at scale, with minimal research costs, and without concerns about human interrater reliability. This text analysis pipeline—associating all of the text units (e.g., sentences) in a dataset with embedding vectors, automating the creation of a training set, and performing classification to label all text units—has many potential applications in the analysis of psychoactive substance experience reports and in other fields.

It is possible that non-pharmacological differences between drug experiences could have contributed to differences in the visual experience reports we analyzed. First, the relative salience of different aspects of the visual effects could vary according to non-visual influences, such as cognitive alterations. In future work, the analysis method we describe in this study can be extended to non-visual aspects of the psychedelic experience to examine this possibility. Second, contextual factors and population differences also contribute to the experience reports. If different substances are statistically associated with different sets and settings associated with the experience, or different populations of experience report authors, such differences might manifest as variation in visual effects in our results. These challenges are inherent to observational studies conducted with self-reported descriptions of self-administered psychedelic substances. In future work, our analysis method could be applied to research studies in which set, setting, and population are more controlled.

In future work, our method for analyzing the visual subjective effect profiles of psychedelics can be used in conjunction with biochemical and physiological measures like receptor binding affinity, functional activation of receptors, and brain activity. In general, effects of psychedelics on conscious experience and global brain activity cannot be directly reduced to binding affinity measures. Different compounds binding to the same receptor can cause different patterns of neural activations for a variety of reasons, including affinity differences, nonlinear effects, threshold effects, and whether the binding is agonistic or antagonistic. Nonetheless, the method of quantitatively measuring psychedelics’ subjective effects that we describe here can be combined with biochemical, anatomical, physiological, and psychological measures to further investigate the actions of psychedelics in the brain and the biological bases of conscious visual experience.

## Conclusion

Much remains to be understood about how activation levels of different neurotransmitter receptor types/subtypes contribute to visual perception and how psychedelics interact with these classes of receptors to affect subjective experience. Moreover, at the level of neural circuits and brain networks, the mechanisms of action of psychedelic substances are still largely unknown. Even so, patterns of receptor binding affinities or actions on individual neurons are insufficient to fully characterize the perceptual and cognitive effects of psychedelics. A compelling explanation of how psychedelics affect brain activity must not only describe their actions on individual neurons but also how changes in the patterns of activity across populations of neurons are linked to perceptual and cognitive effects.

This latter question remains a mystery, but there are several theories being developed that seek to explain the acute effects of psychedelics on perception and cognition and their long-term effects on personality, worldview, and mental health status (Carhart-Harris and Friston, 2019; Vollenweider and Preller, 2020). Our study contributes to these efforts by highlighting the need to account for the roles that multiple types of neurotransmitter receptors play in brain activity.

Finally, our study suggests that psychedelics can be used as effective tools to study basic questions in psychology and neuroscience, such as how patterns of activity in visual cortex relate to different features of visual experience. Future studies can correlate measures such as receptor binding, brain activity, and phenomenological reports across psychedelic substances to disentangle heterogenous biological contributions to the panoply of conscious visual experience.

## Data Availability

The data analyzed in this study are subject to the following licenses/restrictions: researchers partnering with Erowid Center may access the Erowid Experience Vaults database for research purposes. The Erowid Experience Vaults are publicly viewable at the following URL: https://erowid.org/experiences/. Requests to access these datasets should be directed to research@erowid.org.

## Footnotes

^1^ Erowid.org
