# Supplementary material for: A novel method for quantitative analysis of subjective experience reports: application to psychedelic visual experiences
Source: Front Psychol. 2024 Dec 6;15:1397064. doi: 10.3389/fpsyg.2024.1397064 (PMC11663017; doi:10.3389/fpsyg.2024.1397064)
Supplement: Supplementary file 1 [file Table_1.docx]

| **Erowid Dataset Substance Label** | **Chemical Name** | **Taxonomic Identification** | **Other Identification** |
| --- | --- | --- | --- |
| Cannabis |  | Cannabis sativa; Cannabis indica; Cannabis ruderalis |  |
| MDMA | 3,4-methylenedioxymethamphetamine |  |  |
| LSD | d-lysergic acid diethylamide |  |  |
| Salvia divinorum |  | Salvia divinorum; Salvia splendens |  |
| Mushrooms |  | psilocybin-containing mushrooms of the genera Psilocybe and Panaeolus |  |
| DMT | N,N-dimethyltryptamine |  |  |
| DXM | dextromethorphan hydrobromide |  |  |
| Mushrooms_P. cubensis |  | Psilocybe cubensis |  |
| Cocaine | methyl (1R,2R,3S,5S)-3- (benzoyloxy)-8-methyl-8-azabicyclo[3.2.1] octane-2-carboxylate |  |  |
| Ketamine | 2-(2-chlorophenyl)-2-(methylamino)-cyclohexanone |  |  |
| Morning Glory |  | Ipomoea tricolor |  |
| Amphetamines | amphetamine |  |  |
| Kratom |  | Mitragyna speciosa |  |
| 2C-I | 4-iodo-2,5-dimethoxyphenethylamine |  |  |
| Methamphetamine | d-N-methylamphetamine |  |  |
| Syrian Rue |  | Peganum harmala |  |
| H.B. Woodrose |  | Argyreia nervosa |  |
| Nitrous Oxide | nitrous oxide |  |  |
| 2C-B | 4-bromo-2,5-dimethoxyphenethylamine |  |  |
| 2C-E | 4-ethyl-2,5-dimethoxyphenethylamine |  |  |
| Heroin | diacetylmorphine |  |  |
| Oxycodone | (5a)-4,5-epoxy-14-hydroxy-3-methoxy-17- methylmorphinan-6-one |  |  |
| 5-MeO-DMT | 5-methoxy-N,N-dimethyltryptamine |  |  |
| Alcohol | Ethyl alcohol |  |  |
| Pharms_Tramadol | trans-(+/-)-2-[(dimethylamino)methyl]-1-(3-methoxyphenyl) cyclohexanol |  |  |
| Nutmeg |  | Myristica fragrans |  |
| Pharms_Zolpidem | N,N-6-trimethyl-2-p-tolyl-imidazo[1,2-a]pyridine- 3-acetamide L-(+)-tartrate |  |  |
| Diphenhydramine | 2-(diphenylmethoxy)-N,N-dimethylethylamine |  |  |
| Datura |  | Species of the genus Datura, including ceratocaula, inoxia, metel, and stramonium |  |
| Amanitas_A. muscaria |  | Amanita muscaria |  |
| Hydrocodone | 4,5a-Epoxy-3-methoxy-17-methylmorphinan-6-one |  |  |
| Pharms_Alprazolam | 8-chloro-1-methyl-6-phenyl-4H-[1,2,4]-triazolo[4,3-a] [1,4]benzodiazepine |  |  |
| Caffeine | 3,7-dihydro-1,3,7-trimethyl-1h-purine-2,6-dione |  |  |
| 5-MeO-DiPT | 5-Methoxy-N,N-diisopropyltryptamine |  |  |
| 4-AcO-DMT | 4-acetoxy-N,N-dimethyltryptamine |  |  |
| AMT | alpha-methyltryptamine |  |  |
| Cacti_T. pachanoi |  | Trichocereus pachanoi |  |
| Alcohol_Beer_Wine |  |  | ethyl alcohol in beer or wine |
| Alcohol_Hard |  |  | ethyl alcohol in distilled spirit |
| Pharms_Clonazepam | 5-(2-chlorophenyl)-1,3-dihydro-7-nitro2H-1,4-benzodiazepin-2-one (C15H10ClN3O3) |  |  |
| Kava |  | Piper methysticum |  |
| Pharms_Buprenorphine | 17-(cyclopropylmethyl)-alpha-(1,1-dimethylethyl)-4,5-epoxy- 18,19-dihydro-3-hydroxy-6-methoxy-alpha-methyl-6,14- ethenomorphinan-7-methanol |  |  |
| Codeine | (5alpha,6alpha)-7,8-Didehydro-4,5-epoxy-3-methoxy-17-methylmorphinan-6-ol |  |  |
| 2C-T-7 | 4-(n)-propylthio-2,5-dimethoxyphenethylamine |  |  |
| 25I-NBOMe | 4-iodo-2,5-dimethoxy-N-(2-methoxybenzyl)phenethylamine |  |  |
| Dimenhydrinate | o-benzhydryldimethylaminoethanol 8-chlorotheophyllinate |  |  |
| Methoxetamine | 2-(3-methoxyphenyl)-2-(ethylamino)cyclohexanone; 3-MeO-2-Oxo-PCE; |  |  |
| Pharms_Methylphenidate | alpha-phenyl-2-piperidineacetic acid methyl ester; methyl phenidylacetate |  |  |
| Mimosa tenuiflora |  | Mimosa tenuiflora |  |
| DPT | N,N-dipropyltryptamine |  |  |
| Inhalants |  |  | Gaseous chemicals or volatile solvents inhaled for psychoactive effects, including gasoline, acetone, and glue |
| GHB | gamma-hydroxybutyrate |  |  |
| Modafinil | benzhydrylsulphinylacetamide |  |  |
| Huasca Combo |  |  | Monoamine oxidase inhibitors (MAOI)- and N,N-dimethyltryptamine (DMT)-containing plants (or extracts) taken separately |
| Products_Spice-Like Smoking Blends |  |  | Synthetic cannabinoid products |
| Methylone | 3,4-methylenedioxymethcathinone; 2-methylamino-1-(3,4-methylenedioxyphenyl) propan-1-one |  |  |
| Ayahuasca |  |  | South American brew traditionally made from the B. caapi vine and admixtures such as P. viridis, and/or other N,N-dimethyltryptamine (DMT)-containing plants |
| Pharms_Bupropion | 1-(3-chlorophenyl)-2-[(1,1-dimethyl-ethyl)amino]-1-propanone |  |  |
| Pharms_Gabapentin | 1-(aminomethyl)cyclohexanacetic acid |  |  |
| 2C-T-2 | 4-ethylthio-2,5-dimethoxyphenethylamine |  |  |
| Pharms_Venlafaxine | (+/-)-1-[2-(dimethylamino)-1-(4-methoxyphenyl)ethyl] cyclohexanol |  |  |
| Methadone | 6-dimethylamino-4,4-diphenyl-3-heptanone |  |  |
| Morphine | (5a,6a)-7,8-didehydro-4,5-epoxy-17-methylmorphinan-3,6-diol |  |  |
| 4-Methylmethcathinone | 2-Methylamino-1-(4-methylphenyl)propan-1-one |  |  |
| Tobacco |  | Nicotiana rustica |  |
| MDA | 3,4-methylenedioxyamphetamine |  |  |
| Calea zacatechichi |  | Calea zacatechichi |  |
| Poppies_Opium |  | Papaver somniferum |  |
| Banisteriopsis caapi |  | Banisteriopsis caapi |  |
| Melatonin | N-[2-(5-methoxy-1h-indol-3-yl)ethyl]acetamide |  |  |
| Pharms_Paroxetine | (3S-trans)-3-[(1,3-benzodioxol-5-yl-oxy)methyl]-4-(4- fluorophenyl)piperidine |  |  |
| Crack |  |  | Freebase cocaine |
| Pharms_Quetiapine | 2-[2-(4-dibenzo [b,f ] [1,4]thiazepin-11-yl-1-piperazinyl)ethoxy]-ethanol fumarate |  |  |
| Cannabis_Hash |  |  | Cannabis resin; hashish |
| Lotus_Lily_Nymphaea nouchalia var caerulea |  | Nymphaea nouchali var. caerulea |  |
| 5-MeO-AMT | 5-methoxy-alpha-methyltryptamine |  |  |
| Pharms_Pregabalin | (S)-3-(aminomethyl)-5-methylhexanoic acid |  |  |
| Absinthe |  |  | Distilled spirit infused with herbs including the thujone-containing Artemisia absinthium |
| 25C-NBOMe | 4-chloro-2,5-dimethoxy-N-(2-methoxybenzyl)phenethylamine |  |  |
| Smarts_Phenibut | β-Phenyl-γ-aminobutyric acid |  |  |
| Damiana |  | Turnera aphrodisiaca; Turnera diffusa |  |
| Pharms_Diazepam | 7-chloro-1,3-dihydro-1-methyl-5-phenyl-2H-1,4-benzodiazepin-2-one |  |  |
| 1P-LSD | 1-propionyl-lysergic acid diethylamide |  |  |
| PCP | phencyclidine |  |  |
| Catnip |  | Nepeta cataria |  |
| Valerian |  | Valeriana officinalis |  |
| 4-HO-MET | 4-hydroxy-N,N-ethyl-methyltryptamine |  |  |
| Cacti_T. peruvianus |  | Trichocereus peruvianus |  |
| Pharms_Fentanyl | N-(1-phenethyl-4-piperidyl)propionanilide |  |  |
| 2C-C | 4-chloro-2,5-dimethoxyphenethylamine |  |  |
| 5-MeO-MIPT | 5-Methoxy-N,N-Methylisopropyltryptamine |  |  |
| MDPV | Methylenedioxypyrovalerone |  |  |
| Etizolam | 4-(2-chlorophenyl)-2-ethyl-9-methyl-6H-thieno[3,2-f][1,2,4]triazolo[4,3-a][1,4]diazepine |  |  |
| Pharms_Lorazepam | 7-chloro-5-(2-chlorophenyl)-1,3-dihydro-3-hydroxy- 2H-1,4-benzodiazepin-2-one |  |  |
| JWH-018 | 1-pentyl-3-(1-naphthoyl)indole |  |  |
| Sceletium tortuosum |  | Sceletium tortuosum |  |
| Wormwood |  | Artemisia absinthium |  |
| DOC | 2,5-dimethoxy-4-chloroamphetamine |  |  |
| Pharms_Sertraline | (1S-cis)-4-(3,4-dichlorophenyl)-1,2,3,4-tetrahydro- N-methyl-1-naphthalenamine |  |  |
| Mescaline | 3,4,5-trimethoxy-ß-phenethylamine |  |  |
| Brugmansia |  | Species of the genus Brugmansia, including arborea, candida, and sanguinea |  |
| Piracetam | 2-oxo-1-pyrrolidineacetamide |  |  |
| Huasca Brew |  |  | Brews containing monoamine oxidase inhibitors (MAOI)- and N,N-dimethyltryptamine (DMT)-containing plants (or extracts) |

**Supplementary Table 1. Association of Erowid experience report database with specific chemical names, taxonomic names, or other identifiers**
